# Loss of Nek11 Prevents G2/M Arrest and Promotes Cell Death in HCT116 Colorectal Cancer Cells Exposed to Therapeutic DNA Damaging Agents

**DOI:** 10.1371/journal.pone.0140975

**Published:** 2015-10-26

**Authors:** Sarah R. Sabir, Navdeep K. Sahota, George D. D. Jones, Andrew M. Fry

**Affiliations:** 1 Department of Molecular and Cell Biology, University of Leicester, Leicester LE1 9HN, United Kingdom; 2 Department of Cancer Studies, University of Leicester, Leicester LE2 7LX, United Kingdom; Institut de Génétique et Développement de Rennes, FRANCE

## Abstract

The Nek11 kinase is a potential mediator of the DNA damage response whose expression is upregulated in early stage colorectal cancers (CRCs). Here, using RNAi-mediated depletion, we examined the role of Nek11 in HCT116 WT and p53-null CRC cells exposed to ionizing radiation (IR) or the chemotherapeutic drug, irinotecan. We demonstrate that depletion of Nek11 prevents the G2/M arrest induced by these genotoxic agents and promotes p53-dependent apoptosis both in the presence and absence of DNA damage. Interestingly, Nek11 depletion also led to long-term loss of cell viability that was independent of p53 and exacerbated following IR exposure. CRC cells express four splice variants of Nek11 (L/S/C/D). These are predominantly cytoplasmic, but undergo nucleocytoplasmic shuttling mediated through adjacent nuclear import and export signals in the C-terminal non-catalytic domain. In HCT116 cells, Nek11S in particular has an important role in the DNA damage response. These data provide strong evidence that Nek11 contributes to the response of CRC cells to genotoxic agents and is essential for survival either with or without exposure to DNA damage.

## Introduction

Colorectal cancer (CRC) is the third most commonly diagnosed cancer in the Western world. Current standard care for CRC patients following surgery involves chemotherapy combinations that usually include DNA damaging agents. For example, many patients receive FOLFIRI as first line therapy, a combination of folinic acid, 5-fluorouracil (5-FU) and irinotecan [1]. 5-FU is a pyrimidine analogue that blocks DNA synthesis through inhibiting DNA polymerase, while folinic acid potentiates the effect of 5-FU by inhibiting thymidylate synthase. Irinotecan is an inhibitor of topoisomerase I that causes single-strand DNA breaks, which are usually then converted into double-strand breaks (DSBs). These activate the DNA damage checkpoints and cause arrest of the cell cycle at G1/S or G2/M.

The DNA damage response (DDR) is a complex network of cellular processes that lead to multiple outcomes including DNA repair, cell cycle arrest, senescence and apoptosis [2, 3]. The specific outcome is determined by many factors, including the level and type of damage, and integrity of different DDR pathways. The success of DNA damaging agents in cancer treatment relies upon the increased sensitivity of rapidly cycling cancer cells that have weakened DDR pathways. These differences to normal cells provide the therapeutic window required for efficacy. However, the current selection of these agents based predominantly on tumour type is associated with significant toxicity and a better understanding of what factors dictate the response to these drugs would lead to more refined and personalized treatments.

DDR pathways are initiated through activation of ATM or ATR in response to DSBs, stalled replication forks or changes in chromatin structure associated with DNA adducts [2, 3]. To initiate cell cycle arrest, these kinases phosphorylate downstream targets including Chk1, Chk2 and p53. Phosphorylation of p53 leads to its stabilization and increased expression of its transcriptional target, p21. Chk1 and Chk2 phosphorylate and inactivate the Cdc25 phosphatase through promoting its degradation or cytoplasmic sequestration. Together, increased expression of p21 and loss of Cdc25 function block the activation of Cdks necessary for G1/S and G2/M transitions. However, this represents a small snapshot of what are now understood to be highly complex pathways that involve many other enzymatic, regulatory and structural components.

One set of proteins that are beginning to emerge as important regulators of the DDR are the NIMA-related, or NEK, protein kinase family [4]. This family is composed of eleven members of which at least four, Nek1, Nek8, Nek10 and Nek11, have suspected roles in the DDR [5–10]. Nek11 was the first of these to be implicated when its kinase activity was found to be elevated in cells exposed to DNA damaging agents and replication inhibitors [9]. Moreover, this activity is lost upon addition of the ATM/ATR inhibitor, caffeine, suggesting that Nek11 acts downstream of ATM or ATR. More recent mechanistic studies revealed that Nek11 is activated through phosphorylation on Ser-273 by Chk1 upon exposure of cells to ionizing radiation (IR) [7]. Activated Nek11 is capable of phosphorylating Cdc25A on sites within a phosphodegron that promotes recruitment of β-TrCP. This, in turn, leads to ubiquitin-mediated degradation of Cdc25A and cell cycle arrest [11–13]. However, others have argued that the phosphorylation-dependent degradation of Cdc25 is mediated by alternative kinases, such as casein kinase 1 [14, 15]. Nevertheless, Nek11 has also been reported to be a potentially relevant cancer biomarker as elevated Nek11 expression was detected in a set of colorectal adenomas [16]. We therefore set out to test whether Nek11 is required for the response of CRC cells to clinically relevant DNA damaging agents, as well as seek additional evidence for a role for Nek11 in the DDR.

## Results

### Nek11 is required for IR-induced G2/M arrest of HCT116 cells

**Fig 1:**
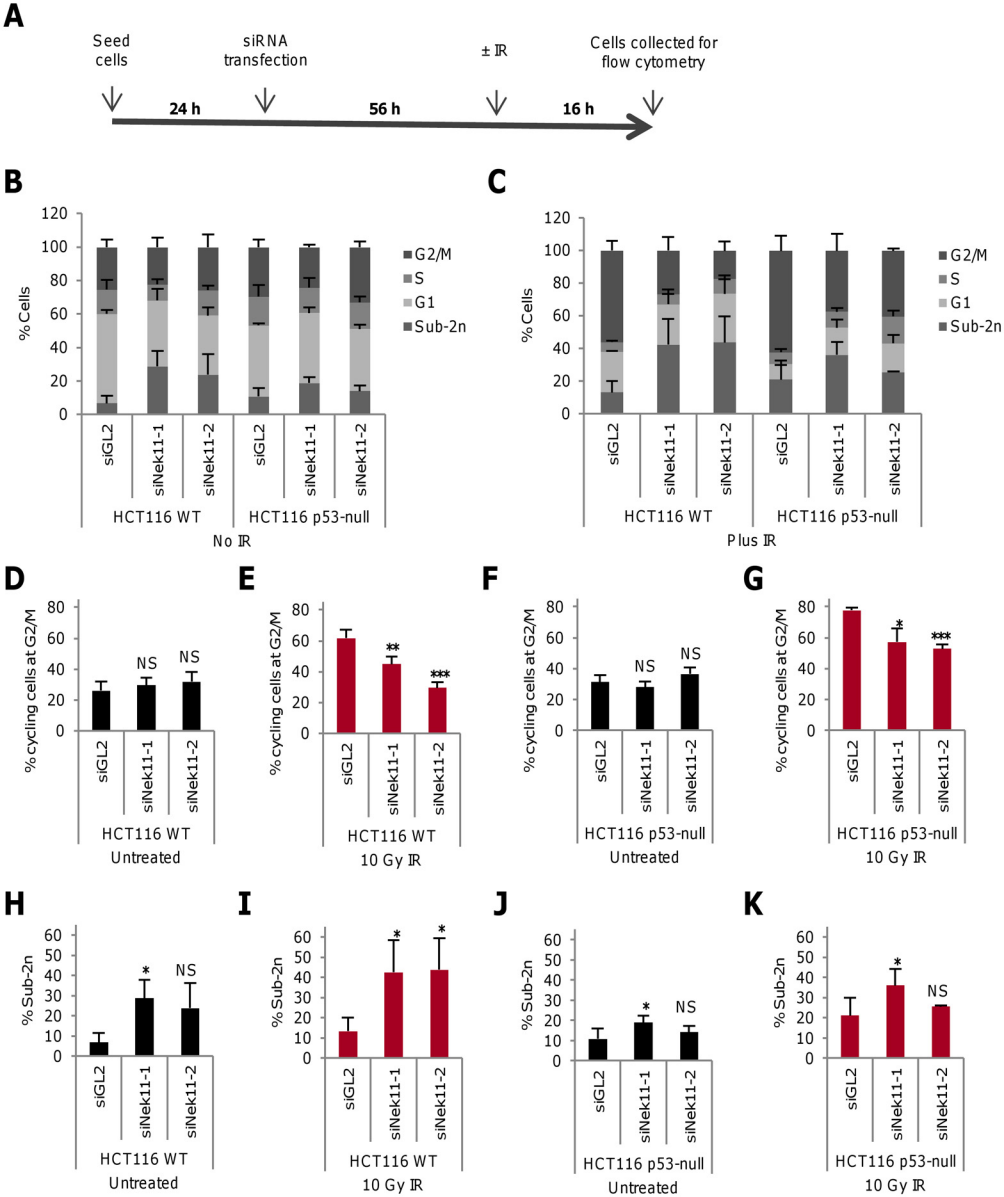
Nek11 depletion leads to loss of G2/M arrest in irradiated HCT116 cells. **A**. Schematic representation of time-course for cell treatments. 24 hours after seeding, cells were transfected with siRNA oligonucleotides. 56 hours later, cells were either untreated or irradiated (±IR). They were then collected and fixed for PI-based flow cytometry after a further 16 hours. **B & C**. Following the protocol described in A, HCT116 WT and p53-null cells were transfected with siRNAs as indicated and left untreated (B) or treated with 10 Gy IR (C), before analysis by flow cytometry. Distribution of cells according to flow cytometry profile is indicated (2n, G1; 2n-4n, S; 4n, G2/M). **D-G.** Histograms represent percentage of cycling HCT116 WT (D, E) and p53-null (F, G) cells at G2/M. **H-K.** Histograms show the percentage of all HCT116 WT (H, I) and p53-null (J, K) cells with sub-2n DNA. Histograms in D-K are taken from data shown in B and C. *p* values are calculated relative to siGL2.

To explore how Nek11 might contribute to the DDR of CRC cells, a protocol was established that allowed cell cycle progression to be monitored by flow cytometry following Nek11 depletion and IR exposure (Fig 1A). Nek11 was depleted using one of two distinct siRNAs with the efficacy of these oligonucleotides confirmed following 72 hours transfection by RT-PCR and Western blot (S1A and S1B Fig). Using a dose range of IR, it was determined that the HCT116 CRC cell line exhibited a major increase in the G2/M fraction 16 hours after exposure with 10 Gy IR (S1C Fig). Hence, in these experiments, cells were first transfected with control (GL2 luciferase) or Nek11 siRNAs and then, after 56 hours, they were either untreated or exposed to 10 Gy IR. Following a further 16 hours, they were collected for analysis by propidium iodide (PI)-based flow cytometry (Fig 1B and 1C, S1D, S1E, S2A and S2B Figs).

Using this protocol, no significant change was observed in the fraction of cycling cells in the G2/M phase of the cell cycle after Nek11 depletion without IR (~30%; Fig 1D). However, following IR exposure, cells depleted of Nek11 exhibited a substantial reduction in the G2/M fraction as compared to cells depleted with control oligonucleotides, with siNek11-2 causing a return to the basal level of G2/M cells (Fig 1E). We note that siNek11-2 gave a more robust knockdown than siNek11-1 by RT-PCR and Western blot. To examine the role of p53 in this response, the same experimental approach was applied to isogenic HCT116 p53-null (p53^-/-^) cells. Depletion of Nek11 alone again had no significant effect on cell cycle distribution in the absence of IR, while there was a marked reduction in G2/M arrest in response to IR treatment following Nek11 depletion (Fig 1F and 1G). However, in this case, neither siRNA caused a complete return to basal levels of G2/M cells suggesting that the loss of G2/M checkpoint control in the absence of Nek11 is partly p53-dependent.

As well as allowing cell cycle distribution to be determined, the flow cytometry analysis revealed the presence of cell death as indicated by the sub-2n peak. Comparison of the percentage in this fraction (relative to all cells in the sample) revealed a modest increase in cell death upon Nek11 depletion without IR, although significance (p<0.05) was only reached with one oligonucleotide (Fig 1H). However, cell death increased to a greater extent in the Nek11 depleted samples following IR exposure suggesting that combined treatment enhanced cell death (Fig 1I). In contrast, there was only a small increase in the sub-2n population of HCT116 p53-null cells following Nek11 depletion before IR exposure and, although there were more cells in the sub-2n fraction following IR exposure, there was not a consistent increase upon Nek11 depletion (Fig 1J and 1K). We therefore conclude that the induction of cell death that results from combined Nek11 loss and IR exposure is largely dependent on p53.

### Nek11 is required to prevent apoptosis and promote long-term cell survival

**Fig 2:**
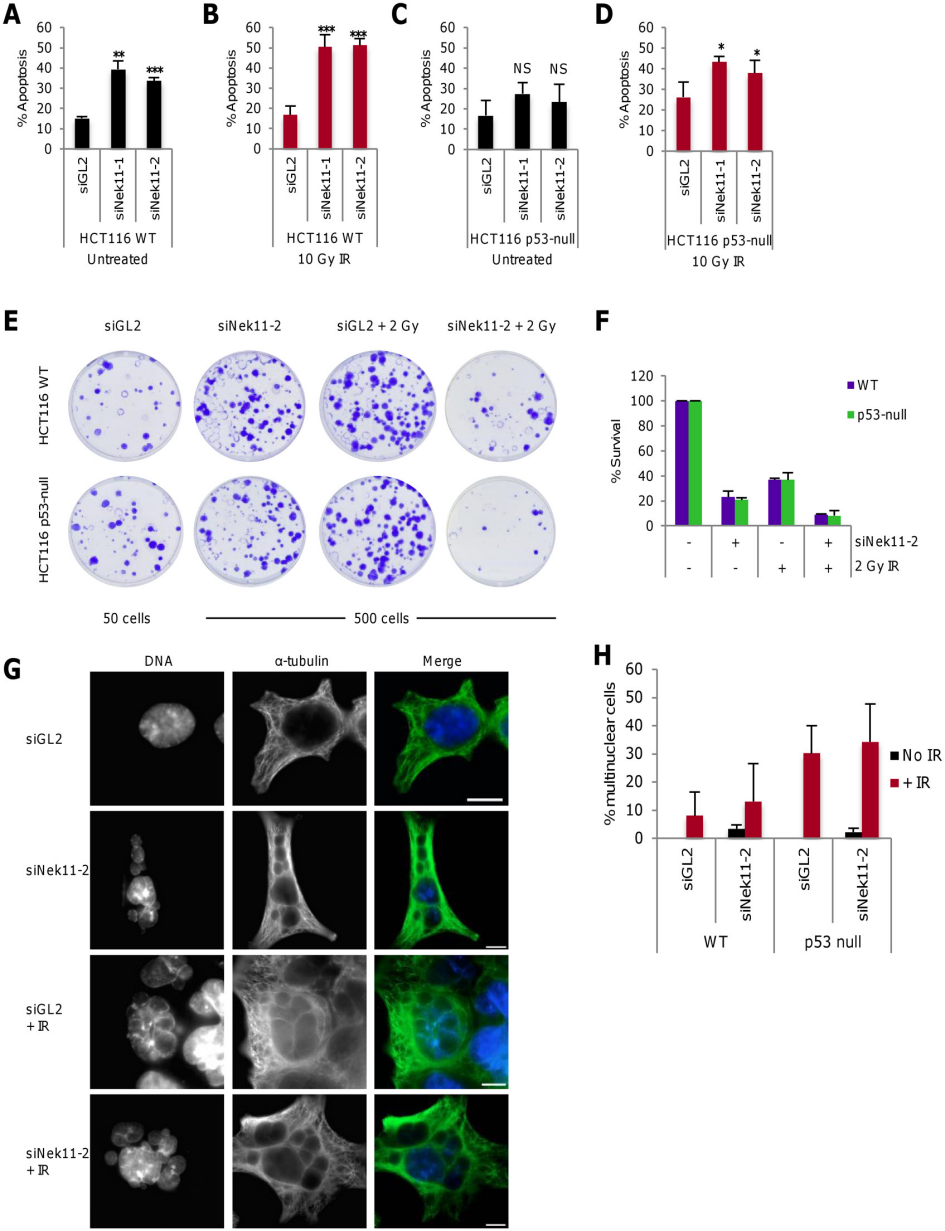
Nek11 depletion leads to apoptosis and loss of cell viability. **A-D.** Following the protocol described in Fig 1A, HCT116 WT (A, B) and p53-null (C, D) cells were transfected with siRNAs as indicated, before irradiation and analysis by annexin V-based flow cytometry. Histograms represent the percentage of all cells in apoptosis. *p* values are relative to siGL2. **E.** HCT116 WT and p53-null cells were transfected with siRNAs as indicated and after 56 hours treated ±2 Gy IR. Cells were collected after a further 16 hours and plated for clonogenic assays (50 cells per plate for siGL2, 500 cells per plate for other treatments). Colonies were stained with crystal violet. **F.** Colonies from E were counted and % survival determined as described in Materials and Methods. **G.** HCT116 p53-null cells were transfected with either siGL2 or siNek11-2 and after 56 hours either left untreated or irradiated. Cells were fixed 48 hours post IR and stained with α-tubulin antibody (green). DNA was stained with Hoechst to observe nuclear morphology. Scale bars, 10 um. **H.** Histogram represents the percentage of cells exhibiting multiple nuclei following the treatments indicated as described in G. 500 cells were counted per sample and the average percentage of multinuclear cells was determined across two independent experiments.

As PI-based flow cytometry indicated cell death following Nek11 depletion, with or without IR exposure, we decided to specifically measure apoptosis. For this, the same protocol was followed as before except that flow cytometry was performed using annexin V-based staining to measure the loss of plasma membrane phospholipid asymmetry that arises during apoptosis. Depletion of Nek11 without IR exposure led to a ~2-3-fold increase in apoptosis in HCT116 WT cells confirming that Nek11 promotes survival in the absence of DNA damage (Fig 2A). Moreover, while exposure to 10 Gy IR alone did not increase the percentage of HCT116 WT cells undergoing apoptosis, there was an enhancement in the apoptotic fraction following combined Nek11 depletion and IR exposure compared to Nek11 depletion alone (Fig 2B). In the HCT116 p53-null cells, Nek11 depletion alone did not cause a significant increase in apoptosis, while there was only a small increase in apoptosis in HCT116 p53-null cells when Nek11 depletion was combined with IR (Fig 2C and 2D). These data are consistent with those obtained using PI-based staining and indicate that loss of Nek11 leads to a p53-dependent induction of apoptosis that is exacerbated by IR exposure.

To examine long-term survival, clonogenic assays were performed in which cells were depleted with control or Nek11 siRNAs for 56 hours and then exposed to a lower dose (2 Gy) of IR before plating out 16 hours later. After two weeks, colonies were fixed and counted (Fig 2E and 2F). First of all, this demonstrated that even in control depleted samples, this dose of IR was sufficient to cause substantially reduced (~3-fold) colony number not only in the HCT116 WT cells, but also the HCT116 p53-null cells. Hence, although IR does not induce substantial cell death or apoptosis at short times after exposure, it does cause long-term loss of survival that occurs in a p53-independent manner. Interestingly, depletion of Nek11 alone also had a strongly deleterious effect on survival with a 5-fold reduction in colony number in HCT116 WT cells. Again, a similar decrease in colony number was seen with the HCT116 p53-null cells. The combination of Nek11 depletion and IR exposure led to the greatest loss of viability with ~10-fold reduction in colony number in both the HCT116 WT and p53-null cells. Therefore, we conclude that long-term survival of HCT116 cells is dramatically reduced in response to IR exposure, Nek11 depletion or a combination of both. Moreover, these responses are not dependent on p53, indicating that p53-independent death pathways are activated.

To examine whether the long-term loss of cell survival following these treatments might result from mitotic catastrophe, mock or Nek11-depleted HCT116 cells were either untreated or exposed to IR and then fixed for microscopic analysis after a further 48 hours. The presence of large multinucleated cells indicative of failed mitotic divisions (i.e. mitotic catastrophe) were seen in response to either Nek11 depletion or IR exposure (Fig 2G). However, quantification of multinucleated cells revealed that depletion of Nek11 alone led to a relatively small frequency of mitotic catastrophe, whereas a substantially higher frequency was observed in response to IR treatment alone (Fig 2H). Similar results were observed in both WT and p53-null cells except that mitotic catastrophe in response to IR was even more pronounced in the p53-null cells consistent with previous reports [17, 18]. The high levels of mitotic catastrophe induced by IR alone suggest that this is a major cause of the reduced viability seen in the clonogenic assays. However, the lower levels of mitotic catastrophe induced by depletion of Nek11 alone rather argue in favour of alternative mechanisms of reduced colony formation, such as damage-induced senescence.

### Nek11 mediates G2/M arrest in HCT116 cells treated with irinotecan

**Fig 3:**
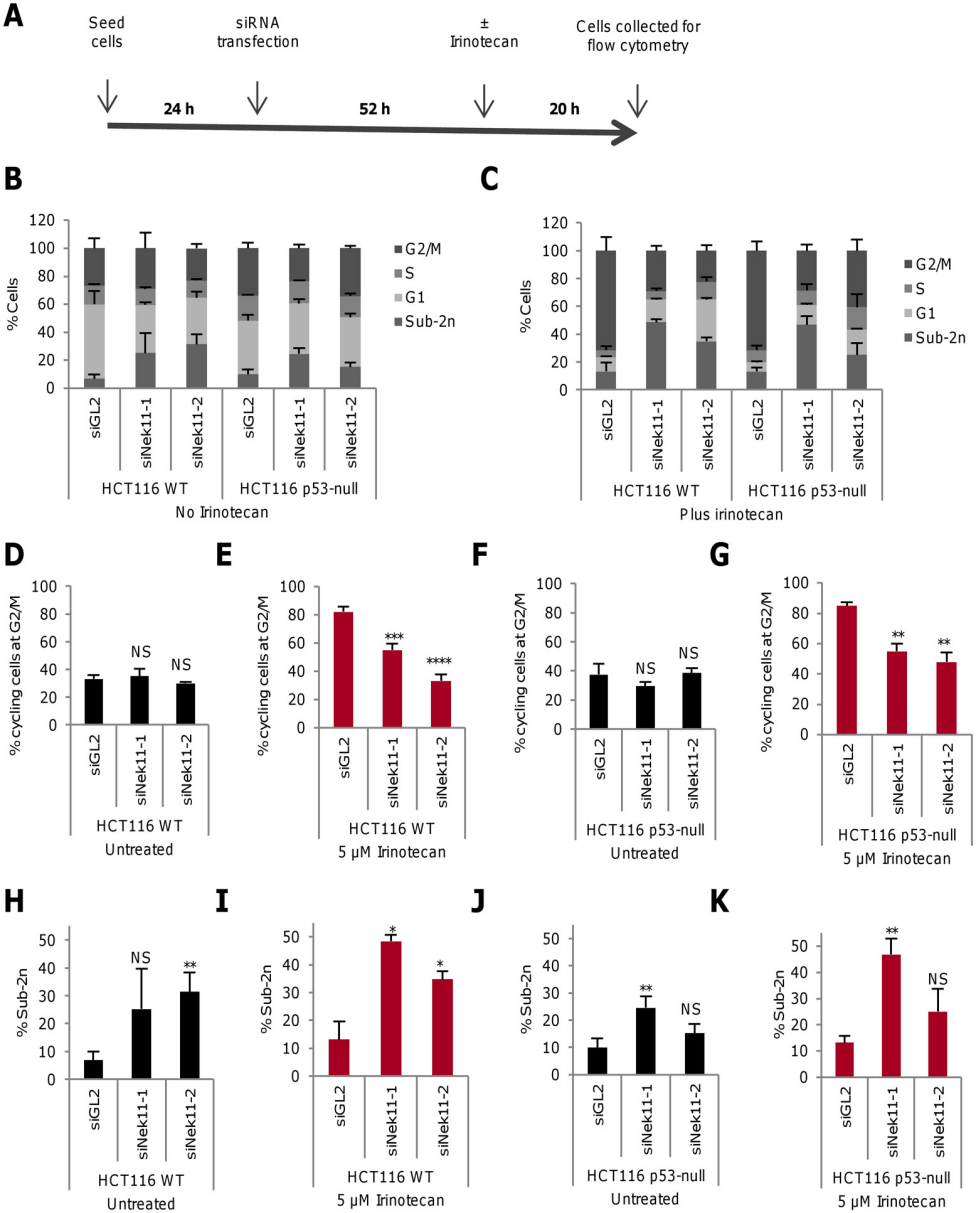
Irinotecan induces Nek11-dependent G2/M arrest in HCT116 cells. **A.** Schematic representation of time-course for cell treatments. 24 hours after seeding, cells were transfected with siRNA oligonucleotides. 52 hours later, cells were either untreated or treated with 5 μM irinotecan. They were then collected and fixed for PI-based flow cytometry after a further 20 hours. **B & C**. Following the protocol described in A, HCT116 WT and p53-null cells were transfected with siRNAs as indicated and left untreated (B) or treated with irinotecan (C), before analysis by flow cytometry. Distribution of cells according to flow cytometry profile is indicated (2n, G1; 2n-4n, S; 4n, G2/M). **D-G.** Histograms represent percentage of cycling HCT116 WT (D, E) and p53-null (F, G) cells at G2/M. **H-K.** Histograms show the percentage of HCT116 WT (H, I) and p53-null (J, K) cells with sub-2n DNA. Histograms in D-K are based on data in B and C. *p* values are relative to siGL2.

CRC patients frequently receive irinotecan, a topoisomerase I inhibitor, as part of their chemotherapy treatment. PI-based flow cytometry confirmed that HCT116 cells exhibit a dose response to this DNA damaging agent, with an accumulation of cells in G2/M following 24 hours treatment (S3A Fig). A protocol was therefore used that allowed us to compare the response of HCT116 WT and p53-null cells to irinotecan to that seen for IR. In this case, cells were depleted of Nek11 for 52 hours prior to addition of irinotecan at 5 μM. After a further 20 hours, cells were collected for analysis by flow cytometry (Fig 3A–3C and S2C and S2D Fig). Firstly, this revealed that, as with IR, the accumulation of HCT116 WT cells in G2/M in response to irinotecan was suppressed following Nek11 depletion (Fig 3D and 3E and S3B Fig). Similarly, irinotecan induced a G2/M arrest in HCT116 p53-null cells that was partly suppressed by Nek11 depletion (Fig 3F and 3G and S3C Fig). As for IR exposure, we observed that Nek11 depletion alone induced a modest level of cell death but this was enhanced in combination with irinotecan in HCT116 WT cells (Fig 3H and 3I). In contrast, a lower and less consistent level of cell death was observed in the HCT116 p53-null cells following Nek11 depletion either with or without irinotecan (Fig 3J and 3K). We therefore conclude that irinotecan also induces a Nek11-dependent G2/M arrest that is partly dependent on p53 status in HCT116 cells, and that irinotecan increases cell death when combined with Nek11 depletion in a manner that is p53-dependent. Hence, Nek11 is likely to play a similar role in the response of HCT116 cells to IR and irinotecan.

### Identification of four Nek11 splice variants that undergo nucleocytoplasmic shuttling

**Fig 4:**
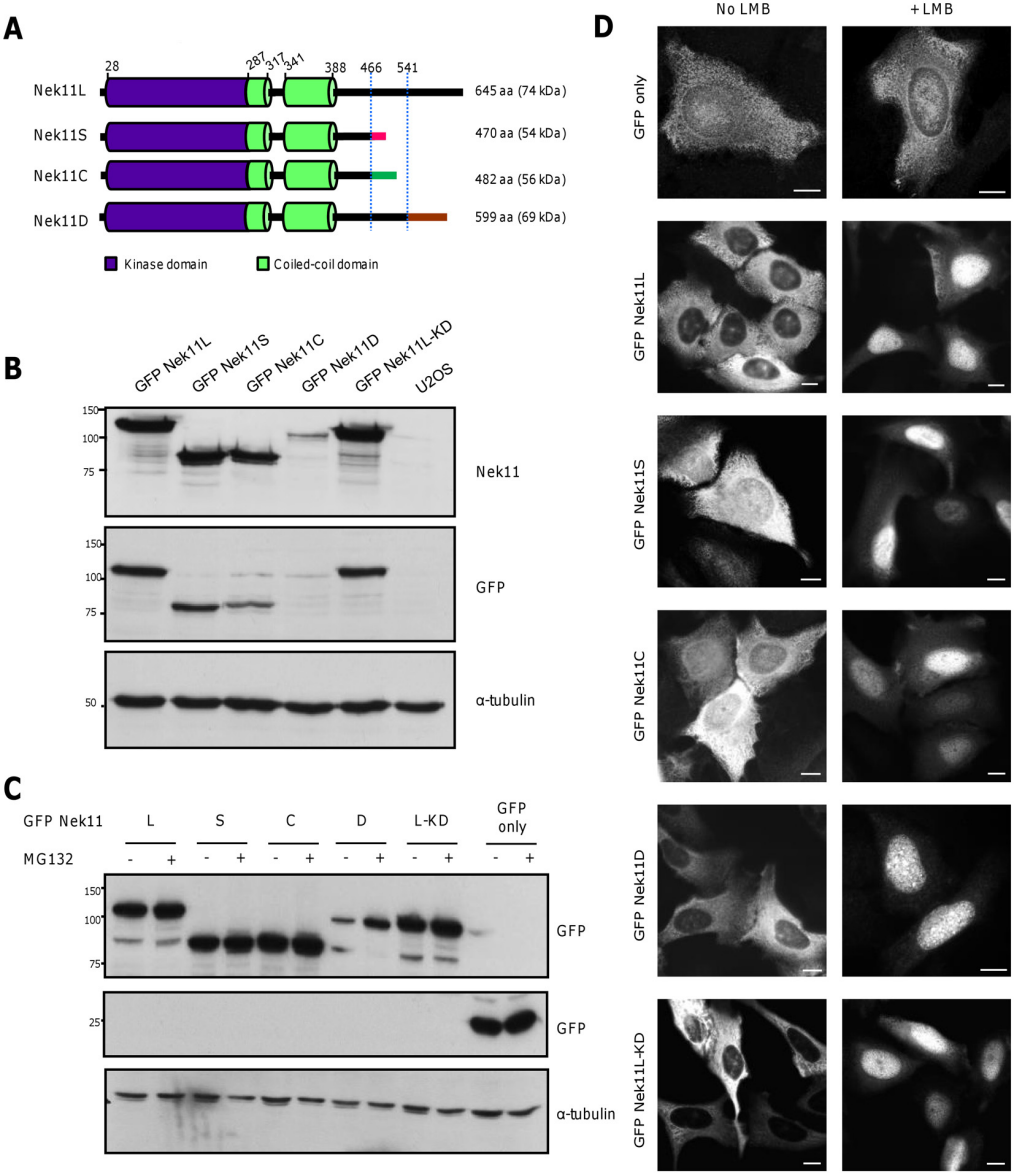
Nek11 splice variants exhibit nucleocytoplasmic shuttling. **A**. Schematic representation of the four Nek11 protein isoforms. The different C-termini are indicated and dotted lines indicate the positions at which the proteins diverge. The positions of kinase domain, coiled-coils, residue numbers and predicted molecular weights are indicated. **B.** Lysates from U2OS:GFP-Nek11 stable cell lines or U2OS parental cells were analysed by SDS-PAGE and Western blotting with antibodies against Nek11, GFP and α-tubulin. **C.** U2OS cells were transfected with constructs indicated and, after 20 hours, treated ±MG132 for 4 hours. Lysates were analysed by Western blot with antibodies indicated. M.wts. (kDa) are indicated on left in B and C. **D.** GFP only and GFP-Nek11 cell lines as indicated were treated ±LMB for 3 hours before fixation and staining with GFP antibodies. Scale bars, 5 μm.

The first characterization of human Nek11 described two splice variants, a long version of 74 kDa, termed Nek11L, and a short version of 54 kDa, termed Nek11S [9]. Through analysis of gene expression databases, we identified two more putative splice variants that we termed Nek11C and Nek11D, with predicted sizes of 56 and 69 kDa, respectively. All four variants have an identical N-terminal sequence of 466 residues that encompasses the catalytic domain and two predicted coiled-coils. Nek11S and Nek11C terminate soon after, albeit with different sequences. Nek11L and Nek11D have more extended C-termini, initially sharing an additional identical ~50 residues before ending with alternative sequences (Fig 4A). To determine whether these variants had distinct properties, we first generated U2OS cells that stably expressed GFP-tagged versions of each Nek11 variant. These cells were used for the purposes of subcellular localization studies, whilst it has been shown that Nek11 depletion perturbs the DDR in these cells [7]. We also generated a U2OS cell line expressing a ‘kinase-dead’ (KD) version of Nek11L with mutation in two essential catalytic residues (K61R/D158A) to determine whether localization might be activity-dependent.

Western blot analysis revealed that the GFP-Nek11D variant was expressed at much reduced levels as compared to the other variants in stable cell lines (Fig 4B). Incubation with the proteasome inhibitor, MG132, led to selective upregulation of this isoform suggesting that the Nek11D variant is unique amongst these four variants in possessing sequences that target it for ubiquitin-mediated degradation, presumably in its unique C-terminus (Fig 4C). Immunofluorescence microscopy revealed that whilst the Nek11L and Nek11D isoforms were restricted to the cytoplasm, Nek11S and Nek11C were detected in both the cytoplasm and nucleus (Fig 4D). Kinase-dead Nek11L was restricted to the cytoplasm like the wild-type protein. As Nek11 was reported to localize to nucleoli [19], we assessed whether the variants might shuttle between the cytoplasm and nucleus. Inhibition of nuclear export with the Crm1 inhibitor, leptomycin B (LMB), caused all four Nek11 variants to accumulate in the nucleus, albeit without obvious concentration in nucleoli. Kinase-dead Nek11L also underwent nucleocytoplasmic shuttling indicating that this is not dependent on its enzymatic activity.

### Identification of sequences regulating nucleocytoplasmic shuttling of Nek11

**Fig 5:**
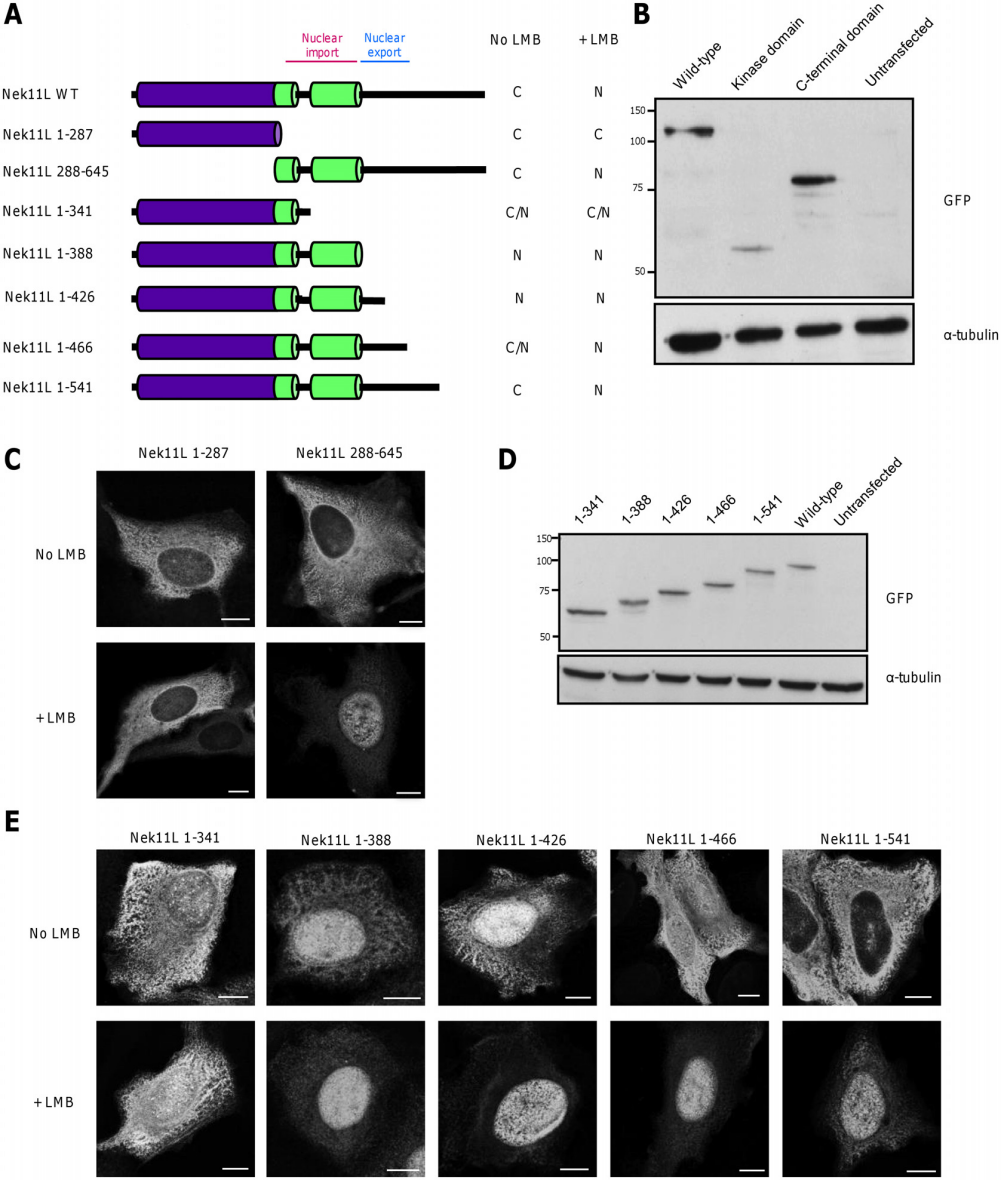
Mapping of regions in Nek11 required for nuclear import and export. **A.** Schematic representation of GFP-Nek11L constructs used to examine subcellular localisation. Predominant localisation to cytoplasm (C), nucleus (N) or equal distribution (C/N) ±LMB treatment is indicated. **B.** Western blots with GFP and α-tubulin antibodies of lysates prepared from U2OS cells transiently transfected for 24 hours with the Nek11L constructs indicated. Kinase domain includes residues 1–287 and C-terminal domain includes residues 288–645. M. wts (kDa) are indicated on the left. **C**. U2OS cells were transfected with constructs indicated and, after 24 hours, treated ±LMB for 3 hours before fixation and staining with GFP antibodies. **D & E**. Western blots and immunofluorescence staining was performed as in B and C, respectively, with the constructs indicated. Scale bars, 5 μm.

To map the regions of the protein required for nuclear import and export, we analysed the localization of a series of Nek11 truncation mutants (Fig 5A). The N-terminal catalytic domain alone (residues 1–287) localized to the cytoplasm and did not shuttle. In contrast, the C-terminal non-catalytic domain of Nek11L (residues 288–645) was cytoplasmic in the absence of LMB but concentrated in the nucleus with LMB. This indicates that it contains motifs necessary for nucleocytoplasmic shuttling (Fig 5B and 5C). As canonical nuclear localization and export sequences were not present, we generated five additional constructs representing C-terminal truncations of Nek11L (Fig 5A and 5D). A construct encompassing the catalytic domain and both coiled-coil motifs (residues 1–388) was nuclear irrespective of LMB treatment, whereas an extended construct encompassing residues 1–541 behaved like full-length protein being cytoplasmic in untreated cells and nuclear with LMB (Fig 5E). A construct that represented the region conserved between all four Nek11 variants, 1–466, exhibited a more equal nuclear-cytoplasm distribution reminiscent of the S and C variants that terminate soon after this point. Similar to those variants, this construct concentrated in the nucleus after LMB treatment. Hence, we propose that the coiled-coil regions contain sequences necessary for nuclear import, whilst the region between residues 388 and 465 contains sequences necessary for nuclear export.

### Nek11S plays a key role in DNA damage-induced G2/M arrest in HCT116 cells

To determine whether all four Nek11 splice variants are expressed in HCT116 cells, as well as three other CRC cell lines (HT29, SW480 and SW620), quantitative RT-PCR was performed with isoform-specific primers (S4A and S4B Fig). These identified mRNAs for each variant in all four cell lines, with Nek11L and Nek11C consistently being the most and least abundant, respectively (S4C Fig). We then compared their abundance in these CRC cell lines relative to that in the immortalized human colonocyte line, HCEC. This indicated notable variation with increased Nek11S in HT29 and increased Nek11C in SW620 (S4D Fig). Otherwise, the levels of each variant were either similar or reduced compared to HCEC cells.

**Fig 6:**
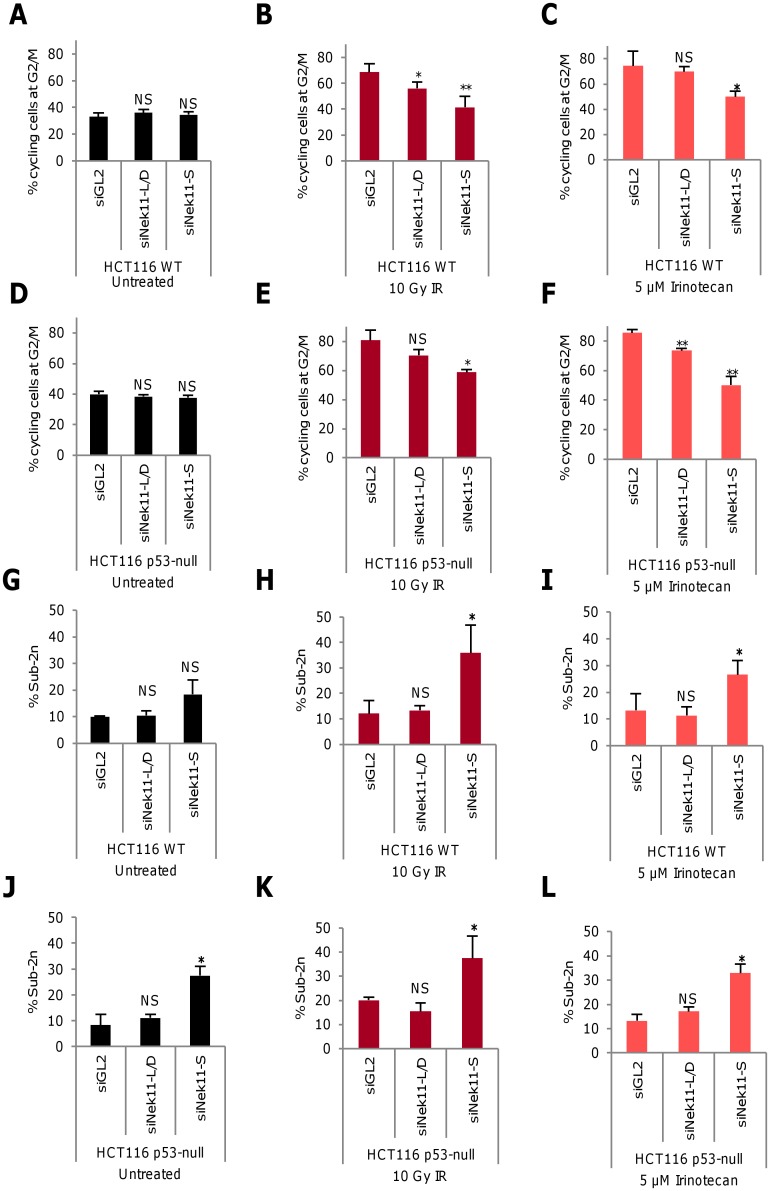
Nek11S is required for G2/M checkpoint arrest and cell survival. **A-L.** Using the protocols described in Fig 1A for irradiation and Fig 3A for irinotecan treatment, HCT116 WT (A-C, G-I) and HCT116 p53-null (D-F, J-L) cells were transfected with siRNA oligonucleotides to co-deplete Nek11L and Nek11D (L/D), or deplete Nek11S or luciferase (siGL2). Histograms show the percentage of cycling cells at G2/M (A-F) and of total cells with sub-2n DNA (G-L). *p* values are relative to siGL2 for each treatment.

To test the contribution of these splice variants to the DDR in HCT116 cells, two sets of siRNAs were validated, one that co-depletes both the longer isoforms, Nek11L and Nek11D, and one that selectively depletes Nek11S (S4E Fig). These were then applied to HCT116 WT and p53-null cells using the protocols described earlier for analysing responses to IR and irinotecan by PI-based flow cytometry (S5 Fig). In HCT116 WT cells, there was a modest reduction in the G2/M population upon depletion of Nek11L/D in response to IR, but not in response to irinotecan. In contrast, there was a substantial reduction in the G2/M population in response to both treatments upon depletion of Nek11S (Fig 6A–6C, S2E and S2F Fig). In the HCT116 p53-null cells, a small but significant reduction in the G2/M population was seen upon Nek11L/D depletion in response to irinotecan but not IR, whereas Nek11S depletion led to a significant reduction in response to both treatments (Fig 6D–6F). As for depletion of total Nek11, it was notable that the fraction of G2/M cells returned to basal levels upon depletion of Nek11S in WT, but not p53-null cells. Hence, although the relative depletion efficiency may vary, these data indicate that at least Nek11S plays an important role in mediating DNA-damage induced G2/M arrest in HCT116 cells, whilst confirming that this response is partly p53-dependent.

Examination of the sub-2n population via PI-based flow cytometry revealed that depletion of Nek11S, but not Nek11L/D, resulted in a significant increase in cell death in HCT116 WT cells exposed to IR or irinotecan (Fig 6G–6I). Depletion of Nek11S, but not Nek11L/D, also led to significant levels of cell death in p53-null cells exposed to IR or irinotecan (Fig 6J–6L). This latter result was unexpected given the previous observations that cell death in Nek11-depleted cells exposed to these treatments is p53-dependent. However, depletion of Nek11S also led to a significant increase in cell death in the absence of genotoxic treatment in p53-null cells suggesting that this was not a specific response to exogenous DNA damage.

## Discussion

Previous studies revealed that the kinase activity of Nek11 is stimulated in HeLa cells exposed to DNA damaging agents and replication inhibitors [9]. Moreover, Nek11 was identified in a screen for genes required for G2/M arrest in U2OS cells exposed to IR, with Nek11 promoting Cdc25A degradation downstream of Chk1 [7]. Here, we show that loss of Nek11 abrogates G2/M arrest and reduces cell survival in HCT116 CRC cells exposed to either IR or the chemotherapeutic agent, irinotecan. Moreover, we show that Nek11 undergoes nucleocytoplasmic shuttling in a manner reminiscent of other DDR proteins. These insights provide further evidence that Nek11 is an important mediator of the G2/M DNA damage response as well as being required for survival of CRC cells.

**Fig 7:**
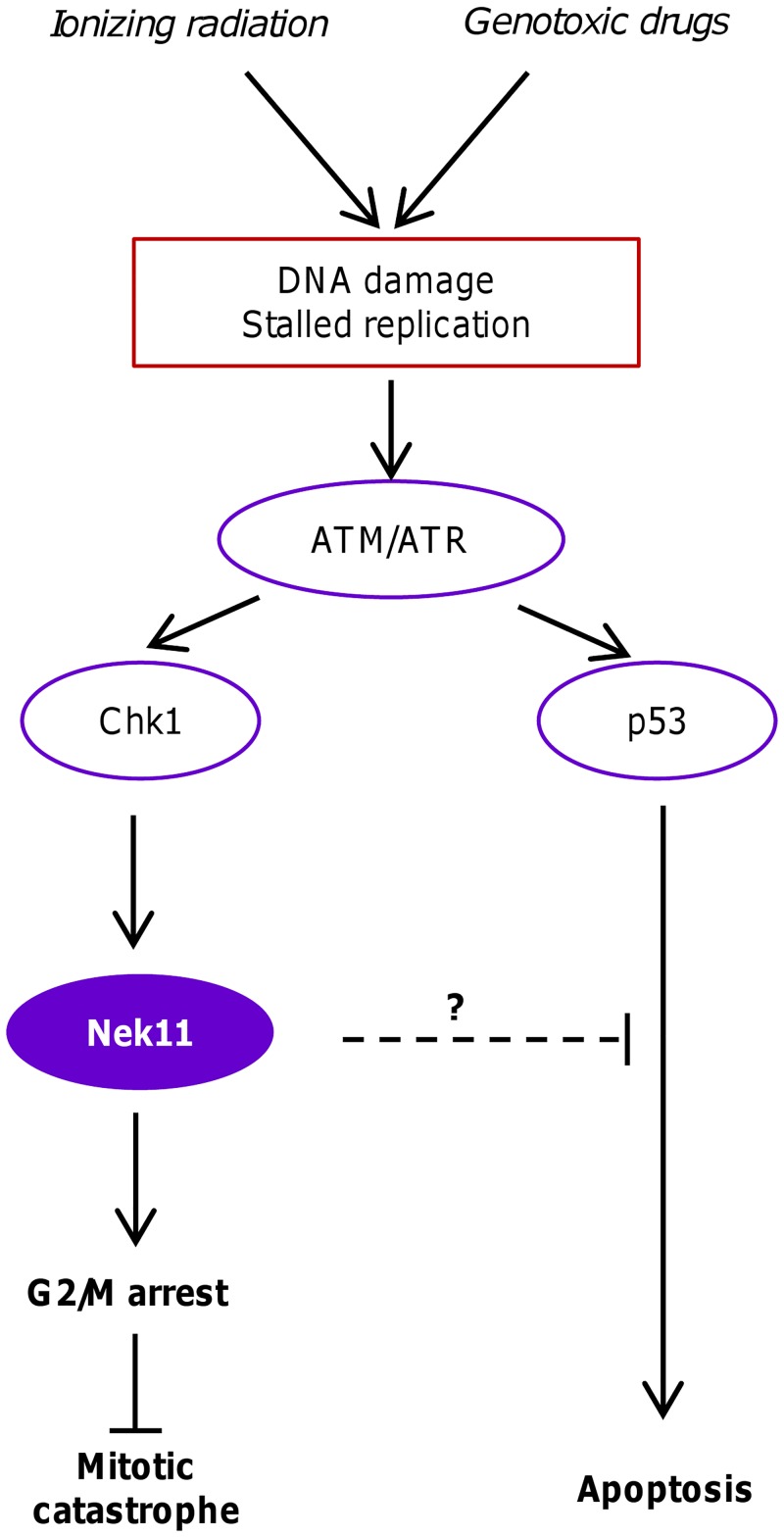
Model for roles of Nek11 in CRC response to genotoxic treatments. This schematic model illustrates the proposed roles of Nek11 in the response of CRC cells to agents that perturb DNA integrity either through direct DNA damage or stalled replication. Previous studies have indicated that Nek11 lies downstream of ATM/ATR and Chk1 and acts to prevent mitotic progression through promoting degradation of Cdc25A. Our results reveal that in CRC cells, Nek11 is required not only to ensure G2/M arrest but also to protect against p53-dependent apoptosis. Failure of the G2/M checkpoint can lead to cell death in a p53-independent manner in part through mitotic catastrophe.

Normal cells exposed to DNA damage arrest primarily at the G1/S transition. However, this checkpoint is often missing in cancer cells that have lost either p53 or Rb. These cells are therefore more reliant on the G2/M checkpoint when exposed to DNA damaging agents. Our studies revealed that while exposure of HCT116 cells to both IR and irinotecan led to a major increase in the G2/M fraction, consistent with activation of the G2/M checkpoint, this fraction was substantially reduced upon removal of Nek11. In the WT cells, Nek11 depletion reduced the G2/M fraction to the baseline level present in a cycling population supporting a potential role for Nek11 in the G2/M checkpoint in HCT116 cells. However, in the p53-null cells, the G2/M fraction, although significantly reduced, remained above baseline. This suggests that Nek11 not only imposes a p53-independent G2/M arrest following DNA damage but, in addition, prevents a p53-dependent loss of G2/M cells (Fig 7).

Consistent with this, we observed a modest increase in the number of cells in the sub-2n fraction, indicative of dying cells, in the Nek11-depleted WT cells exposed to IR or irinotecan that was not seen with the p53-null cells. Likewise, specific analysis of the apoptotic fraction by annexin V assay revealed that a small fraction of Nek11-depleted WT, but not p53-null, cells exposed to IR or irinotecan entered apoptosis. Hence, in the absence of Nek11, some HCT116 cells exposed to exogenous DNA damage undergo a p53-dependent apoptosis, whereas others presumably re-enter the cell cycle in a p53-independent manner. Due to the presence of unrepaired DNA, the likelihood is that these latter cells enter an aberrant mitosis that promotes further genetic damage leading to death either in mitosis or during subsequent cell cycle progression.

When long-term survival responses were analysed by clonogenic assay, it was observed that loss of Nek11 alone was sufficient to substantially impair viability, while this was exacerbated by additional IR exposure. In contrast to the short-term apoptotic response, the loss of long-term viability was not p53-dependent. This fits our model that, without Nek11, cells with DNA damage not only fail to activate a p53-dependent response, but also trigger alternative responses that prevent cell proliferation. We examined whether this was the result of mitotic catastrophe, a process in which cells with damaged DNA progress through mitosis but without undergoing division. This leads to generation of multinucleated cells that trigger cell death by delayed apoptosis or necrosis. Indeed, mitotic catastrophe is recognised to be a major, albeit delayed, response of solid tumours to clinical radiotherapy, occurring several days following irradiation [18]. However, whilst we confirmed that IR triggers mitotic catastrophe and that this is exacerbated by the loss of p53 [19], Nek11 depletion alone only induced a relatively low level of mitotic catastrophe. Hence, whilst it might partly explain the reduced viability, other processes are likely to be involved. Whatever the mechanism, our data indicate that targeted inhibition of Nek11 could inhibit proliferation whether used alone or in combination with DNA damaging agents even in p53-deficient tumours. Whether particular mutations in HCT116 cells make them sensitive, and whether normal cells or other CRC cells are equally reliant on Nek11 for survival are important questions to address. The answers will indicate whether a therapeutic window or mechanistic biomarker can be identified for use with a Nek11 inhibitor.

Two Nek11 splice variants, Nek11L and Nek11S, were previously described [9]. Here, we identify two additional splice variants, Nek11C and Nek11D, and show that all four variants are expressed in CRC cells. By depleting specific isoforms, we attempted to test whether they have differing levels of importance in the HCT116 response to IR or irinotecan. Despite similar levels of knockdown by RT-PCR, we found that loss of Nek11S had a greater consequence on the G2/M arrest than loss of Nek11L and Nek11D. Nek11D, at least as a recombinant protein, is highly unstable so therefore may be less functionally important. Nek11S on the other hand is not only relatively stable but also localizes more efficiently to the nucleus than Nek11L. This could suggest a reason for its greater importance in the DDR. However, the low levels of endogenous Nek11 proteins in HCT116 cells means that we cannot confirm the extent of protein knockdown in these cells and it remains to be determined whether these variants play redundant or distinct roles in the DDR.

We demonstrated that each Nek11 splice variant is capable of nucleocytoplasmic shuttling. Analysis of truncation mutants revealed that the coiled-coils downstream of the catalytic domain are required for nuclear import, whereas a region C-terminal to the second coiled-coil is necessary for nuclear export. Although all four variants contain both targeting motifs, the export sequence does not function as effectively in Nek11LS and Nek11C as these two proteins, as well as a similar-sized truncation mutant, were more evenly distributed between cytoplasm and nucleus. These regions of Nek11 do not contain canonical nuclear localization or export sequences implying that shuttling is mediated through interaction with other proteins that carry targeting motifs. Nucleocytoplasmic shuttling may well be crucial to the role of Nek11 in the G2/M checkpoint in common with other DDR components. For example, Chk1 and Cdc25A are predominantly found in the nucleus but shuttle between the nucleus and cytoplasm [20, 21]. Moreover, DNA damage promotes release of Chk1 from chromatin and its appearance in the cytoplasm [22]. Interestingly, as well as Cdc25A, Nek11 was reported to phosphorylate the Bloom syndrome helicase, BLM, a nuclear protein that is recruited to sites of damaged DNA [23]. This phosphorylation promotes its interaction with TopBP1, a protein required for the replication stress response [24]. A role in the downstream response to both DNA damage and stalled replication is consistent with activation of Nek11 by both DNA damage and replication inhibitors, although whether nuclear entry of Nek11 is necessary for both these functions remains to be determined.

Although DDR proteins have the capacity to act as tumour suppressors, essential components of the replication stress and G2/M checkpoints are not widely inactivated in human cancers. To our knowledge, inactivating mutations in Nek11 have not been identified so far in cancer genome sequencing projects. We chose to focus on CRC due to the demonstration that Nek11 expression is increased in early stage colorectal tumours before being down-regulated in more advanced tumours [16]. One explanation for this could be that Nek11 is upregulated as a protective measure in early stage dysplastic cells, but is then lost in invasive tumours. Our studies in cultured cells suggest that this loss may promote further genome instability. Furthermore, recent studies have linked Nek11 loss with drug resistance in ovarian cancer [25]. Plans to validate the efficacy of a Nek11 inhibitor would therefore have to take account of its expression when selecting an appropriate trial population.

In summary, our data add to the growing weight of evidence that Nek11 is a G2/M checkpoint component. Moreover, they demonstrate an essential role for Nek11 in the viability of HCT116 cells as well as their response to DNA damage. Together, this provides a strong rationale for considering Nek11 as an attractive target for the development of novel DDR-based cancer therapeutics. Further studies are required to investigate whether these may exhibit synthetic lethality with other checkpoint or DNA repair inhibitors or have particular value in cells lacking specific DDR pathways.

## Materials and Methods

### Plasmid construction and mutagenesis

Full-length cDNAs expressing Nek11 isoforms were amplified by RT-PCR from U2OS cells (Nek11S and Nek11D), or obtained from commercial ORFs (Nek11L and Nek11C) and inserted into pLEICS-21 (PROTEX, University of Leicester) for expression with N-terminal GFP tags in mammalian cells. For generation of Nek11L truncations, inserts were amplified from the full-length GFP-Nek11L plasmid and cloned into pLEICS-21. Mutations were introduced using the Quickchange^®^ II XL Site-Directed Mutagenesis Kit according to manufacturer’s instructions (Stratagene). All constructs were confirmed by DNA sequencing (PNACL, University of Leicester).

### Antibody generation

Antibody production was undertaken by Cambridge Research Biochemicals. New Zealand White (NZW) Specific Pathogen Free (SPF) rabbits were housed in a barrier building at Durham University and maintained according to UK Home Office approval as monitored by the Durham University Institutional Animal Care and Use Committee (IACUC). Rabbits were immunized with a bacterially-expressed Nek11 fragment (residues 288–446) fused to an N-terminal His-tag and sera collected from clinically healthy animals. Rabbits were culled with a non-schedule 1 method, being exsanguination via cardiac puncture, as covered by the UK Home Office Project Licence. For affinity purification, antisera were passed over Nek11 immunogen, covalently bound to CNBr-activated sepharose according to manufacturer’s instructions (Amersham), and eluted with 100 mM glycine pH 2.5 and then 100 mM Triethylamine (TEA) pH 11.5 into 1 M Tris-HCL pH 8.0.

### Cell culture and transfection

U2OS, CRC and the immortalized HCEC cell lines were obtained and cultured as described [26–28]. Stable expression was established by transfection into U2OS cells and selection with 1 mg/ml G418 Sulfate (Calbiochem). Resistant cells were pooled. Plasmids were transfected with Lipofectamine 2000 (Invitrogen), while siRNAs were transfected at 100 nM using Oligofectamine™ (Invitrogen). siRNAs (Qiagen) were siNek11-1 (GAACAAGAAUCCUUCAUUA) and siNek11-2 (GAAGGAGGCUGCUCAUAUA) from Dharmacon, and Nek11L/D (CTGCCTATGCTTGGAGTCATA; TGAGATAAGCTTATAGATCAA) Nek11S (CTGGAATAGCCTGAGACTCTA; CTGGGTCTACAAGGAGCATGA) and GL2 (AACGTACGCGGAATACTTCGA).

### Drug treatments and irradiation

Where indicated, HCT116 cells were treated with irinotecan hydrochloride (Sigma) at concentrations stated. U2OS cells were treated with 20 μM MG132 for 4 hours (Calbiochem) or Leptomycin B (LMB) (Calbiochem) at 20 nM for 3 hours where indicated. For irradiation, cells were exposed to X-rays at 1 Gy/min (250 kV constant potential, Pantak X-ray machine).

### Fixed cell microscopy, apoptosis assays and flow cytometry

Fixed cell microscopy and flow cytometry was performed as described [29]. For microscopy, primary antibodies were against GFP (0.5 μg/ml; Abcam) and α-tubuin (0.4 μg/ml; Abcam); DNA was stained with 0.8 μg/ml Hoechst 33258. Apoptosis and cell cycle distributions were determined by Annexin-V and propidium iodide staining, respectively.

### Preparation of cell extracts, SDS-PAGE and Western blotting

Cell lysis, SDS-PAGE and Western blotting were performed as described [30]. Primary antibodies were against Nek11 (0.5 μg/ml; generated as above), GFP (0.2 μg/ml; Abcam) and α-tubulin (0.1 μg/ml; Abcam). Blots were developed by enhanced chemiluminescence (Pierce).

### RNA isolation and qRT-PCR

RNA was isolated using Tri reagent (Sigma), reverse transcribed using Superscript III reverse transcriptase (Invitrogen) and qPCR reactions performed using a Sybr-green master mix (Fermentas). Reactions were performed on LightCycler 480 (Roche) and expression levels calculated as ΔΔC_t_ with GAPDH as the calibrator.

### Clonogenic assays

Cells were seeded (50–2000 cells/well of 6-well plate) and allowed to proliferate until visible colonies were detected (~12–14 days). Plating efficiency for HCT116 WT and HCT116 p53-null cells was 76.2% and 71.3%, respectively. Colonies were fixed with 100% methanol and stained with 0.5% crystal violet solution (Sigma). Mean plating efficiency (PE) of cells from each treatment was calculated as PE = ([average number of colonies counted]/[number of cells plated])x100. The surviving fraction (SF) for each treatment was calculated as SF = ([PE of treated sample]/[PE of control])x100.

### Flow cytometry and microscope image acquisition and processing

Apoptosis was analysed using a Muse Cell Analyzer and Muse 1.3 Analysis software (Merck Millipore). Cell cycle distributions were analysed using a BD FACSCanto™ II analyser and FACSDiva 6.0 software (Becton Dickinson Biosciences). Fluorescence microscopy was performed using a Nikon TE300 inverted microscope using a Plan Apo 60x or 100x DIC oil immersion objective (NA 1.4). Images were obtained using an ORCA-R^2^ camera (Hamamatsu) using Velocity software, v6.0.1 (PerkinElmer), and images processed using Adobe Photoshop 7. Alternatively, microscopy was performed on a Leica TCS SP5 laser scanning confocal microscope equipped with a Leica DMI 6000B inverted microscope. Images were captured and processed using Leica LAS AF software.

### Statistical analysis

Results are mean and standard deviation (S.D.) of three independent experiments. *p* values were calculated using a one-tailed unpaired Student’s t-test assuming unequal variance and represent *, *p*<0.05; **, *p*<0.01; ***, *p*<0.001; ****, *p*<0.0001.

## Supporting Information

S1 Fig Validation of Nek11 depletion and response of HCT116 cells to irradiation. **A**. HCT116 WT cells were transfected with siRNAs indicated, RNA was extracted 72 hours post-transfection and qPCR analysis carried out using Nek11 isoform specific primers. **B.** U2OS cells were transfected with siRNAs indicated, lysed 72 hours post-transfection and analysed by Western blotting with antibodies indicated. Molecular weights are indicated (kDa), together with positions of the Nek11L and D (L/D) and Nek11S and C (S/C) isoforms. **C.** HCT116 WT cells were irradiated with the dose indicated (Gy) and analysed by PI-based flow cytometry after 16 hours. Distribution of cells according to flow cytometry profile is indicated (2n, G1; 2n-4n, S; 4n, G2/M). **D & E**. HCT116 WT (D) and p53-null (E) cells were treated according to the protocol in Fig 1A and analysed by flow cytometry. Data in D and E are presented as composite histograms in Fig 1B and 1C, respectively.
(TIF)

S2 Fig Flow cytometry event plots. Single cell event plots shown as contour maps representing propidium-iodide based flow cytometry data obtained for experiments described in Figs 1A, 1B, 3C, 3D, 6E and 6F.
(TIF)

S3 Fig Flow cytometry analyses of HCT116 cells treated with irinotecan. **A.** HCT116 WT cells were treated with irinotecan at the indicated concentrations and analysed by PI-based flow cytometry after 24 hours. **B & C**. HCT116 WT (B) and p53-null (C) cells were treated according to the protocol in Fig 3A and analysed by flow cytometry. Data in B and C are presented as composite histograms in Fig 3B and 3C, respectively.
(TIF)

S4 Fig RT-PCR analysis of Nek11 splice variants. **A**. Schematic diagram showing the exonic structure of the human Nek11 gene and the four spice variants generated. Red boxes indicate untranslated regions and purple boxes indicate coding region. Red arrows indicate regions to which isoform specific primers were designed for qPCR analysis. **B**. Table of primers used in qPCR experiments with predicted amplification product size. **C.** mRNA was extracted from the cell lines indicated and used for qPCR with Nek11 isoform-specific primers. Histogram shows expression of each isoform on a log scale relative to Nek11C within each cell line. **D.** Samples from C were normalised against GAPDH. The difference in Ct values for CRC cell lines compared to HCEC was calculated and relative expression determined using Q = 2^-ΔΔCt^. **E.** HCT116 WT cells were transfected with siRNAs against luciferase (siGL2) or the Nek11L and D isoforms, (siNek11L/D) or Nek11S (siNek11S), and mRNA abundance determined by qPCR analysis with isoform-specific primers. Histogram shows expression of each isoform relative to siGL2.
(TIF)

S5 Fig Nek11S is required for G2/M arrest in HCT116 cells exposed to DNA damage. **A & B**. HCT116 WT (A) and p53-null (B) cells were transfected with siRNAs indicated and processed according to the protocols in Fig 1A for untreated and IR and Fig 3A for irinotecan. Full flow cytometry profiles based on PI-based staining are shown. **C-E.** Histograms represent percentage of cells in Sub-2n, G1, S and G2/M phases for experiments undertaken as described in A and B. Distributions for untreated (C), irradiated (D) and irinotecan-treated (E) cells are shown.
(TIF)
