# Supplementary figures and images for: Loss of Nek11 Prevents G2/M Arrest and Promotes Cell Death in HCT116 Colorectal Cancer Cells Exposed to Therapeutic DNA Damaging Agents
Source: PLoS One. 2015 Oct 26;10(10):e0140975. doi: 10.1371/journal.pone.0140975 (PMC4621075; doi:10.1371/journal.pone.0140975)

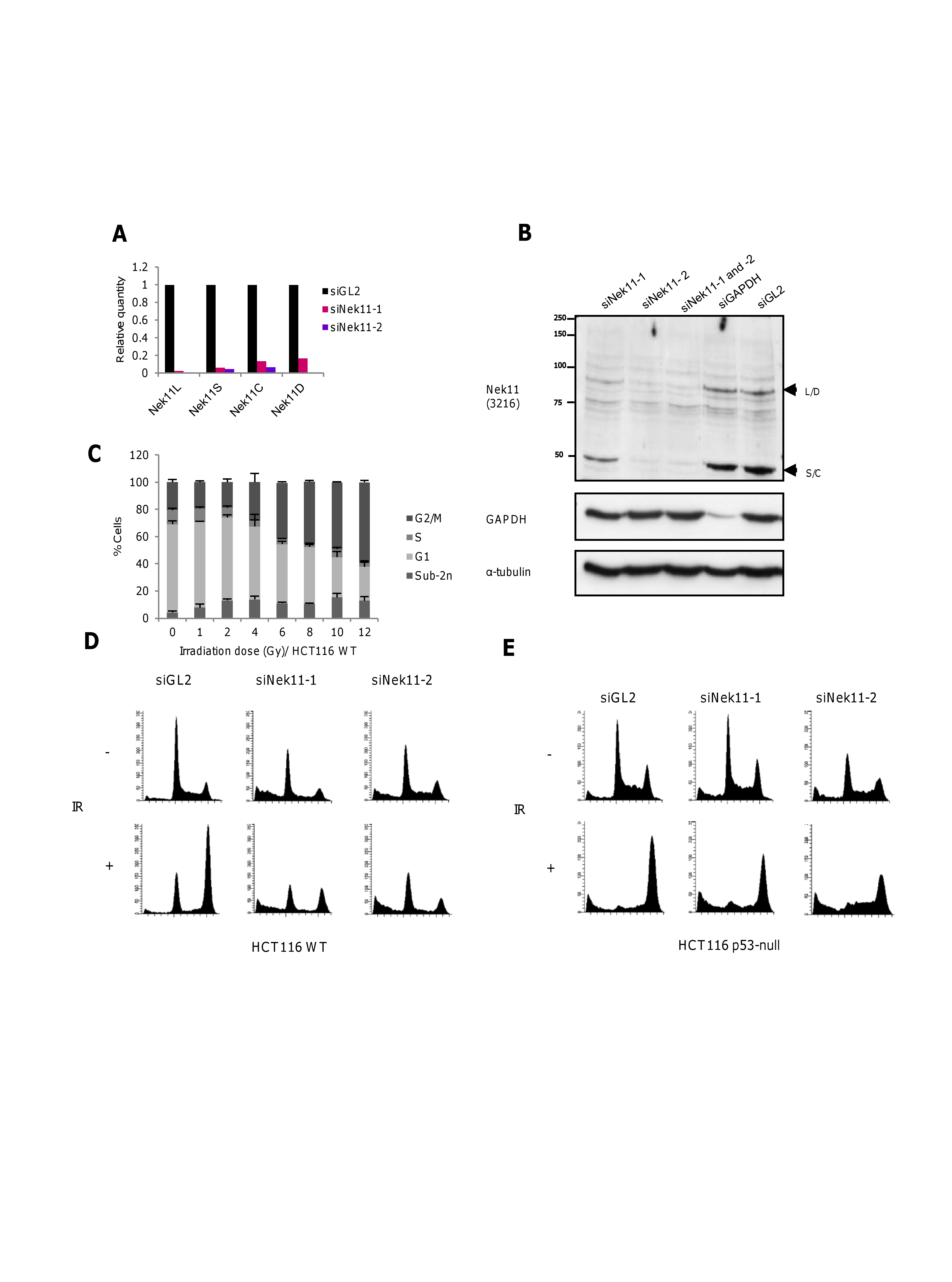

Supplement: S1 Fig — A. HCT116 WT cells were transfected with siRNAs indicated, RNA was extracted 72 hours post-transfection and qPCR analysis carried out using Nek11 isoform specific primers. B. U2OS cells were transfected with siRNAs indicated, lysed 72 hours post-transfection and analysed by Western blotting with antibodies indicated. Molecular weights are indicated (kDa), together with positions of the Nek11L and D (L/D) and Nek11S and C (S/C) isoforms. C. HCT116 WT cells were irradiated with the dose indicated (Gy) and analysed by PI-based flow cytometry after 16 hours. Distribution of cells according to flow cytometry profile is indicated (2n, G1; 2n-4n, S; 4n, G2/M). D & E. HCT116 WT (D) and p53-null (E) cells were treated according to the protocol in Fig 1A and analysed by flow cytometry. Data in D and E are presented as composite histograms in Fig 1B and 1C, respectively. (TIF) [file pone.0140975.s001.tif]

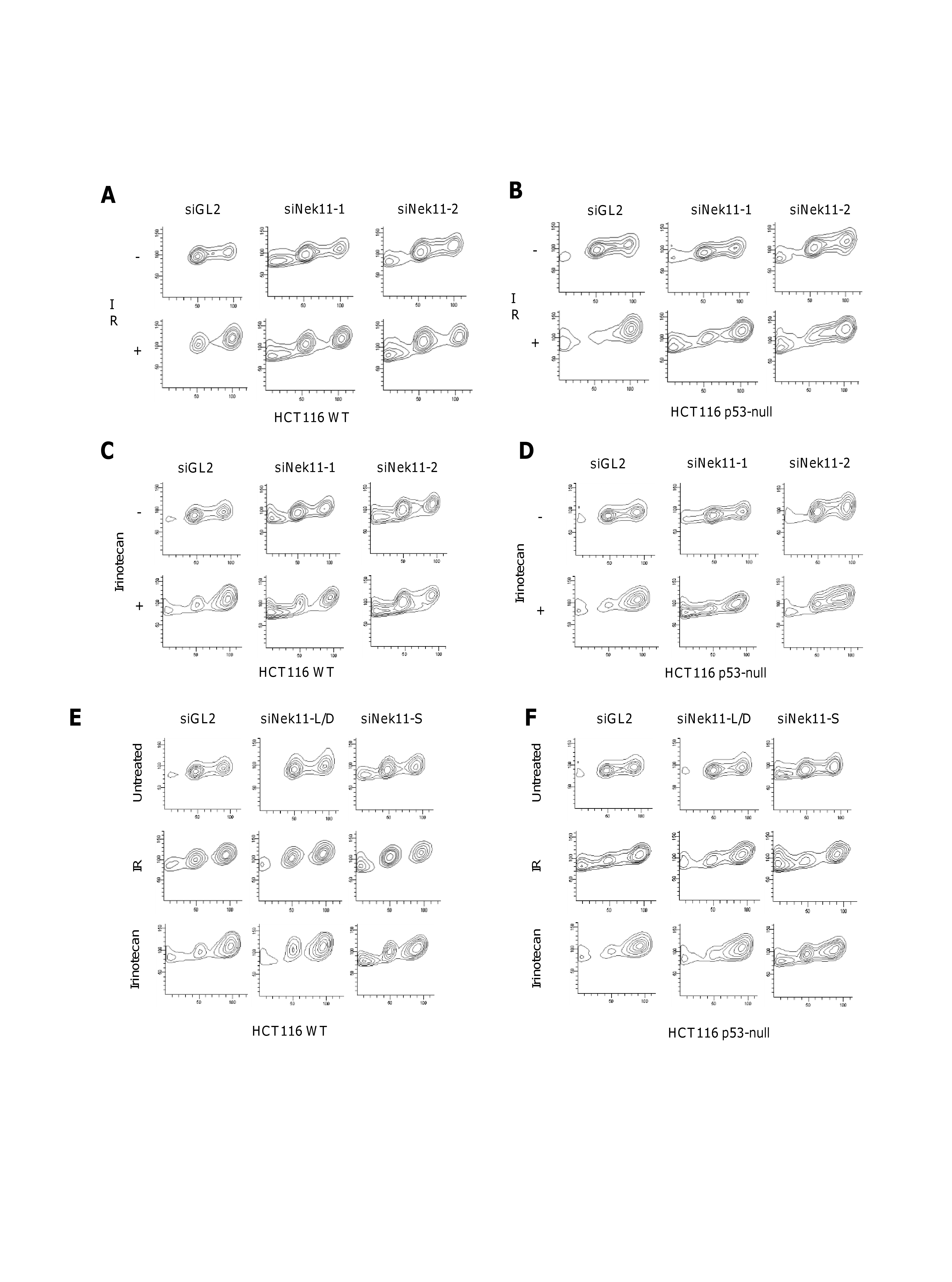

Supplement: S2 Fig — Single cell event plots shown as contour maps representing propidium-iodide based flow cytometry data obtained for experiments described in Figs 1A, 1B, 3C, 3D, 6E and 6F. (TIF) [file pone.0140975.s002.tif]

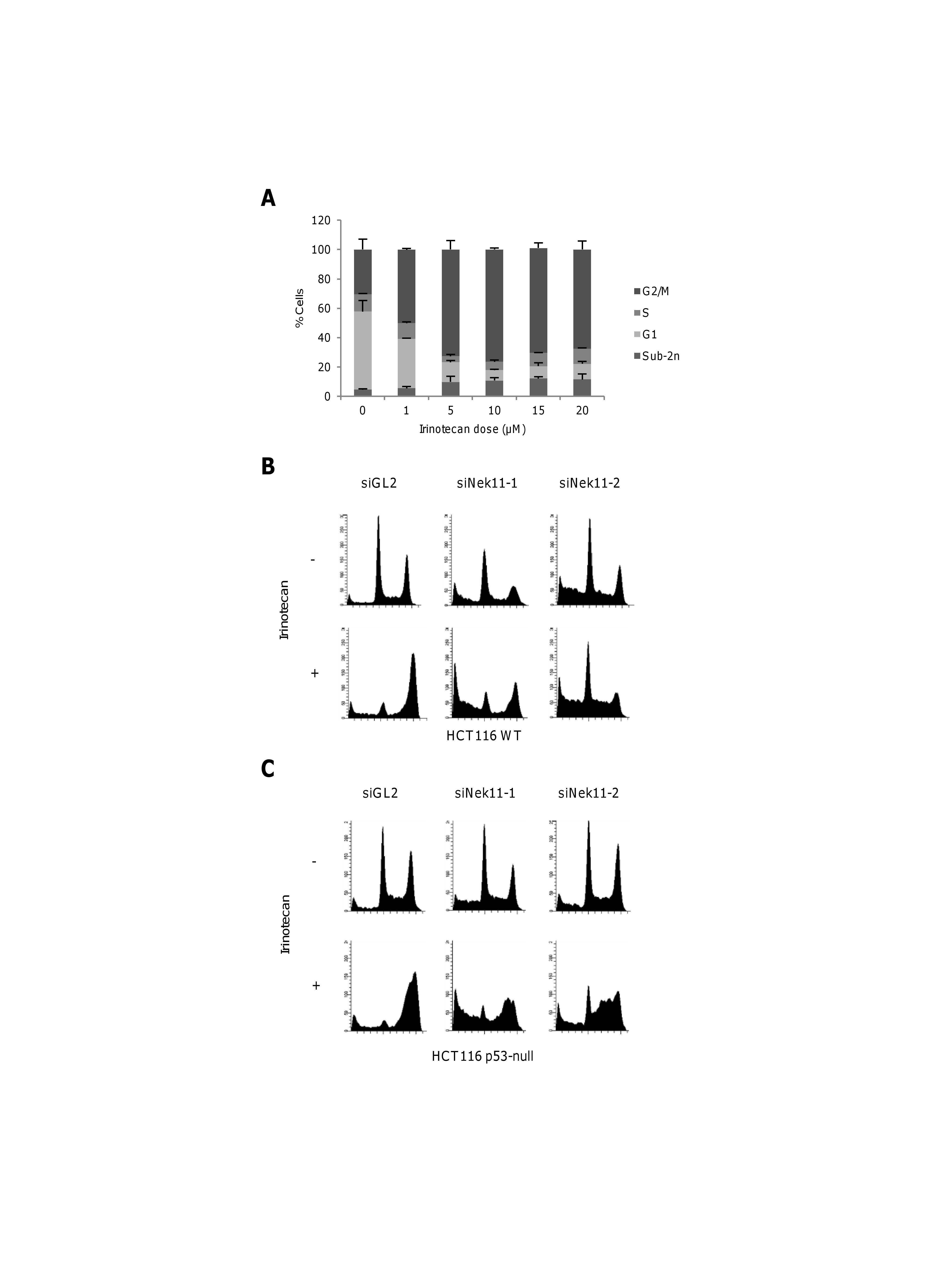

Supplement: S3 Fig — A. HCT116 WT cells were treated with irinotecan at the indicated concentrations and analysed by PI-based flow cytometry after 24 hours. B & C. HCT116 WT (B) and p53-null (C) cells were treated according to the protocol in Fig 3A and analysed by flow cytometry. Data in B and C are presented as composite histograms in Fig 3B and 3C, respectively. (TIF) [file pone.0140975.s003.tif]

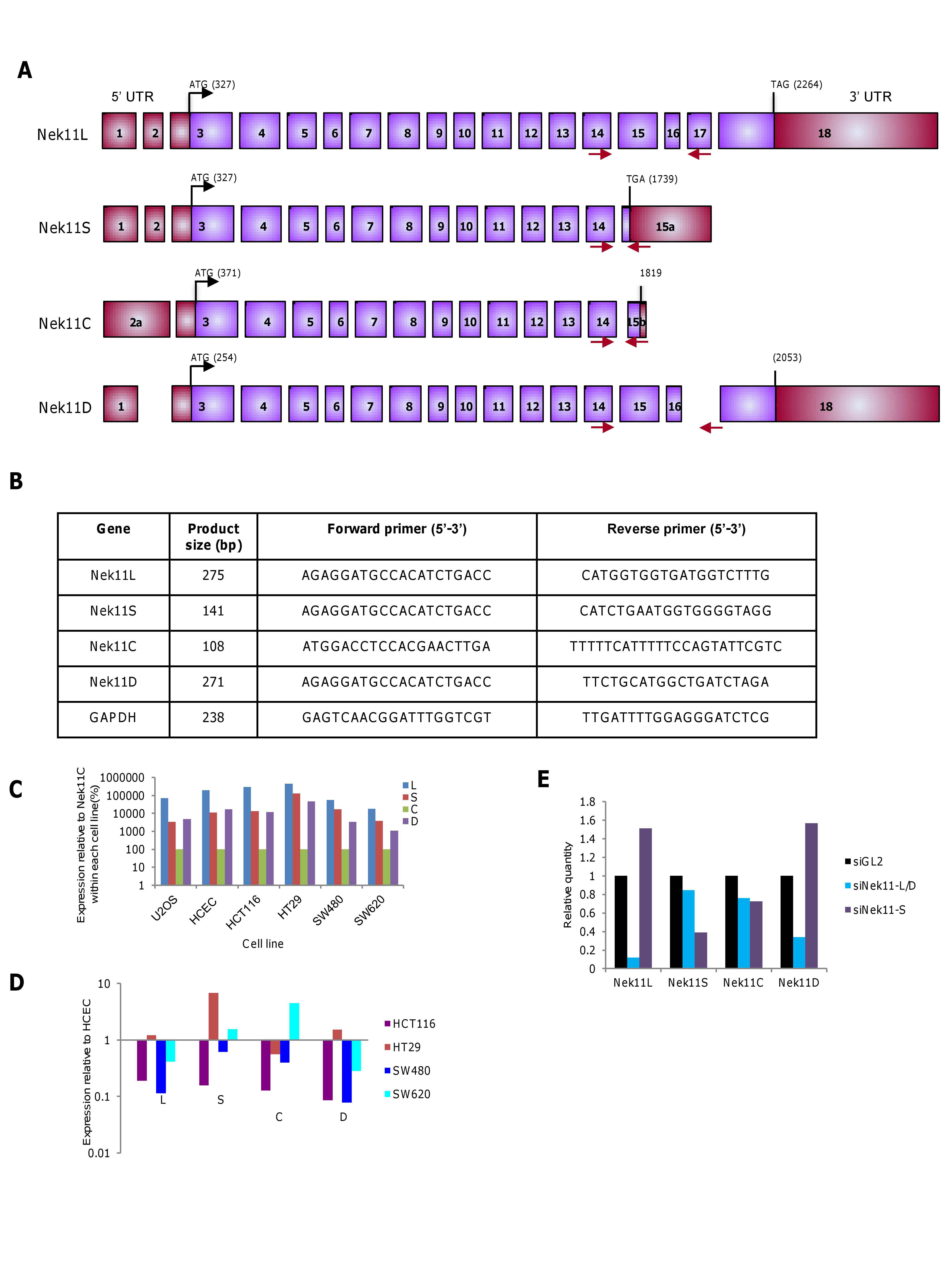

Supplement: S4 Fig — A. Schematic diagram showing the exonic structure of the human Nek11 gene and the four spice variants generated. Red boxes indicate untranslated regions and purple boxes indicate coding region. Red arrows indicate regions to which isoform specific primers were designed for qPCR analysis. B. Table of primers used in qPCR experiments with predicted amplification product size. C. mRNA was extracted from the cell lines indicated and used for qPCR with Nek11 isoform-specific primers. Histogram shows expression of each isoform on a log scale relative to Nek11C within each cell line. D. Samples from C were normalised against GAPDH. The difference in Ct values for CRC cell lines compared to HCEC was calculated and relative expression determined using Q = 2-ΔΔCt. E. HCT116 WT cells were transfected with siRNAs against luciferase (siGL2) or the Nek11L and D isoforms, (siNek11L/D) or Nek11S (siNek11S), and mRNA abundance determined by qPCR analysis with isoform-specific primers. Histogram shows expression of each isoform relative to siGL2. (TIF) [file pone.0140975.s004.tif]

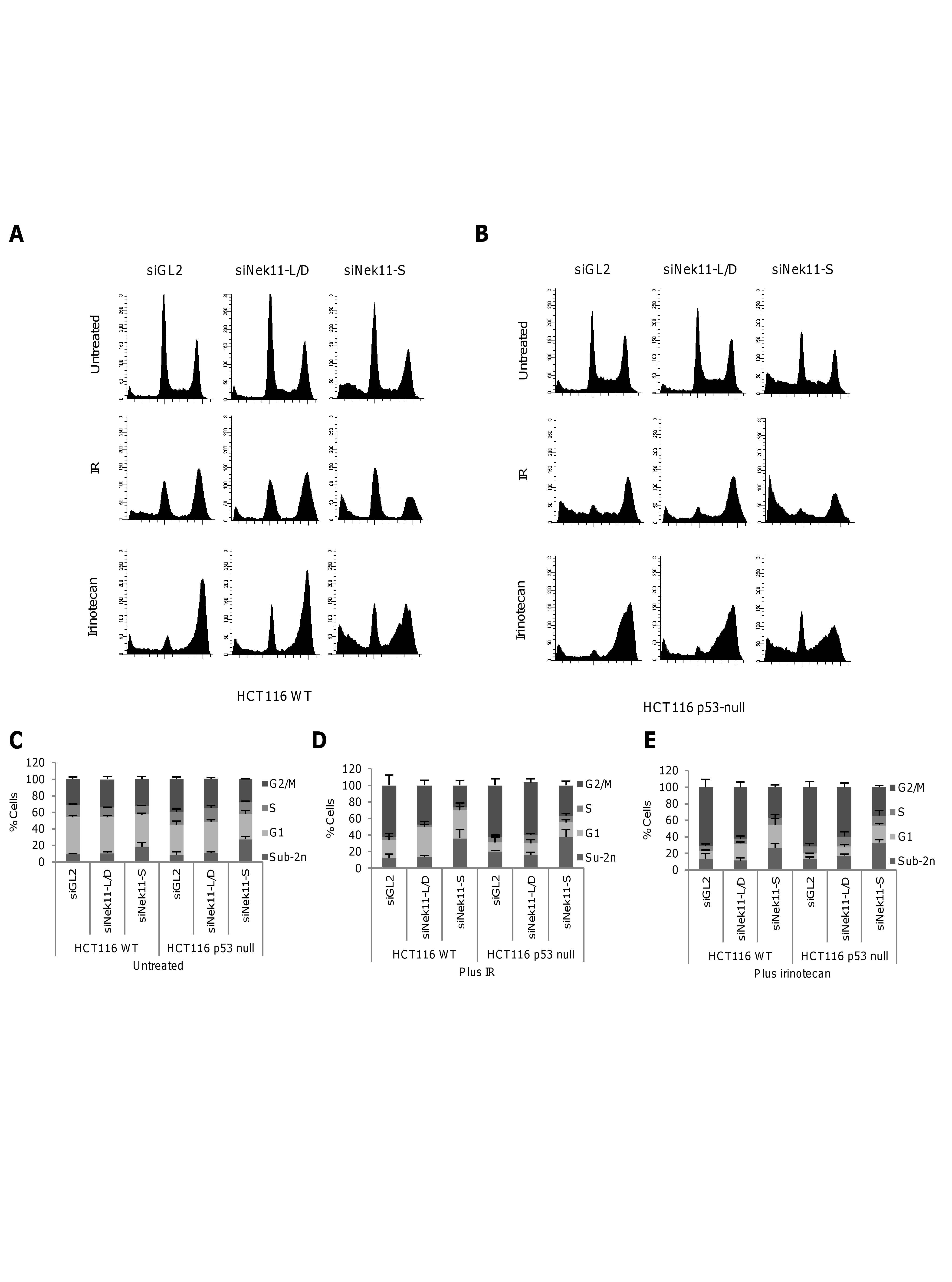

Supplement: S5 Fig — A & B. HCT116 WT (A) and p53-null (B) cells were transfected with siRNAs indicated and processed according to the protocols in Fig 1A for untreated and IR and Fig 3A for irinotecan. Full flow cytometry profiles based on PI-based staining are shown. C-E. Histograms represent percentage of cells in Sub-2n, G1, S and G2/M phases for experiments undertaken as described in A and B. Distributions for untreated (C), irradiated (D) and irinotecan-treated (E) cells are shown. (TIF) [file pone.0140975.s005.tif]
